# Structural studies of *Pseudomonas* and *Chromobacterium* ω-aminotransferases provide insights into their differing substrate specificity

**DOI:** 10.1107/S0907444912051670

**Published:** 2013-03-14

**Authors:** Christopher Sayer, Michail N. Isupov, Aaron Westlake, Jennifer A. Littlechild

**Affiliations:** a Henry Wellcome Building for Biocatalysis, Biosciences, College of Life and Environmental Sciences, University of Exeter, Stocker Road, Exeter EX4 4QD, England

**Keywords:** aminotransferases, transaminases, substrate specificity, industrial biocatalysis

## Abstract

The crystal structures and inhibitor complexes of two industrially important ω-aminotransferase enzymes from *Pseudomonas aeruginosa* and *Chromobacterium violaceum* have been determined in order to understand the differences in their substrate specificity. The two enzymes share 30% sequence identity and use the same amino acceptor, pyruvate; however, the *Pseudomonas* enzyme shows activity towards the amino donor β-alanine, whilst the *Chromobacterium* enzyme does not. Both enzymes show activity towards *S*-α-methylbenzylamine (MBA), with the *Chromobacterium* enzyme having a broader substrate range. The crystal structure of the *P. aeruginosa* enzyme has been solved in the holo form and with the inhibitor gabaculine bound. The *C. violaceum* enzyme has been solved in the apo and holo forms and with gabaculine bound. The structures of the holo forms of both enzymes are quite similar. There is little conformational difference observed between the inhibitor complex and the holoenzyme for the *P. aeruginosa* aminotransferase. In comparison, the crystal structure of the *C. violaceum* gabaculine complex shows significant structural rearrangements from the structures of both the apo and holo forms of the enzyme. It appears that the different rigidity of the protein scaffold contributes to the substrate specificity observed for the two ω-­aminotransferases.

## Abstract 2

The X-ray structures of two ω-aminotransferases from *P. aeruginosa* and *C. violaceum* in complex with an inhibitor offer the first detailed insight into the structural basis of the substrate specificity of these industrially important enzymes.

## 1. Introduction   

The aminotransferases (ATs; transaminases; EC 2.6.1.–) catalyse the transfer of an amino group from an amino acid to a keto acid (Mehta *et al.*, 1993 ▶). They use the cofactor pyridoxal 5′-phosphate (PLP), the biologically active form of vitamin B_6_, which is one of nature’s most versatile cofactors (Braunstein & Shemyakin, 1953 ▶; Metzler *et al.*, 1954 ▶). The mechanism of ATs has been well studied both enzymatically and structurally. Most ATs have high affinity for the cofactor, which usually binds to the enzyme in an internal aldimine form in which C4′ of PLP forms a Schiff base with the NZ atom of the active-site lysine. The amino group of the donor substrate forms a Schiff base with the cofactor during the first half-reaction (external aldimine). After a number of further intermediate steps, including a proton-abstraction step, the amino group is transferred to the cofactor to produce enzyme-bound pyridoxamine 5′-phosphate (PMP) and a keto acid. In the second half-reaction an amino group is transferred from PMP to an acceptor keto acid, producing an amino acid and restoring the PLP internal aldimine.

The application of enzymes in ‘white biotechnology’ for the synthesis of industrially important chiral compounds is becoming increasingly important in the pharmaceutical industry. The use of AT enzymes for the production of optically pure amines and amino alcohols is of key importance in the synthesis of many important drugs such as (*S*)-rivastigmine used in the treatment of dementia resulting from Alzheimer’s and Parkinson’s diseases (Emre *et al.*, 2004 ▶; Fuchs *et al.*, 2010 ▶), (*S*)-repaglinide used in the treatment of type 2 diabetes (Plosker & Figgitt, 2004 ▶) and (*R*)-levocetirizine, which is an antihistamine (Chen, 2008 ▶). Recently, collaboration between Merck and Codexis has resulted in the development of an *R*-specific mutant AT that has been used for the synthesis of sitagliptin, the active ingredient in Januvia^TM^, which is used for the treatment of type 2 diabetes (Savile *et al.*, 2010 ▶). This enzymatic route for the production of sitagliptin resulted in higher enantioselectivity and yields, and was awarded the 2010 Greener Reaction Conditions Prize (http://www.epa.gov/greenchemistry/pubs/pgcc/winners/grca10.html). This award recognized the scientific innovation for sustainable chemistry to produce a drug of significant importance to health using a biocatalytic approach. Most industrial AT substrates are not α-amino acids; therefore, there is increasing interest in the AT enzymes which are capable of catalysing reactions using substrates without an α-­carboxyl group.

The ATs belonging to class III as defined by the Pfam classification (Punta *et al.*, 2012 ▶) are collectively referred to as ω-amino-acid ATs (ωATs; Malik *et al.*, 2012 ▶). They catalyse the transamination of ω-amino acids such as β-alanine or γ-­aminobutyric acid in which the transferred amino group is not adjacent to the carboxyl group. Some enzymes from this class display activity towards substrates without a carboxyl group. Diamine:ketoglutarate AT was the first enzyme reported to show such activity (Kim, 1964 ▶; Samsonova *et al.*, 2003 ▶). A transamination reaction at an ω-carbon position is significantly more difficult to catalyse than that at an α-carbon position. Therefore, most α-amino-acid ATs (classes I, II, IV and V) are unable to catalyse a reaction on substrates without an α-carboxyl group. For instance, serine:pyruvate AT from *Sulfolobus solfataricus* (class V; Sayer *et al.*, 2012 ▶) is very active towards phenylalanine but shows no activity towards the corresponding amino alcohol (phenylalaninol) or the β-­amino-acid analogue 3-phenyl-3-aminopropionate.

**Figure 1:**
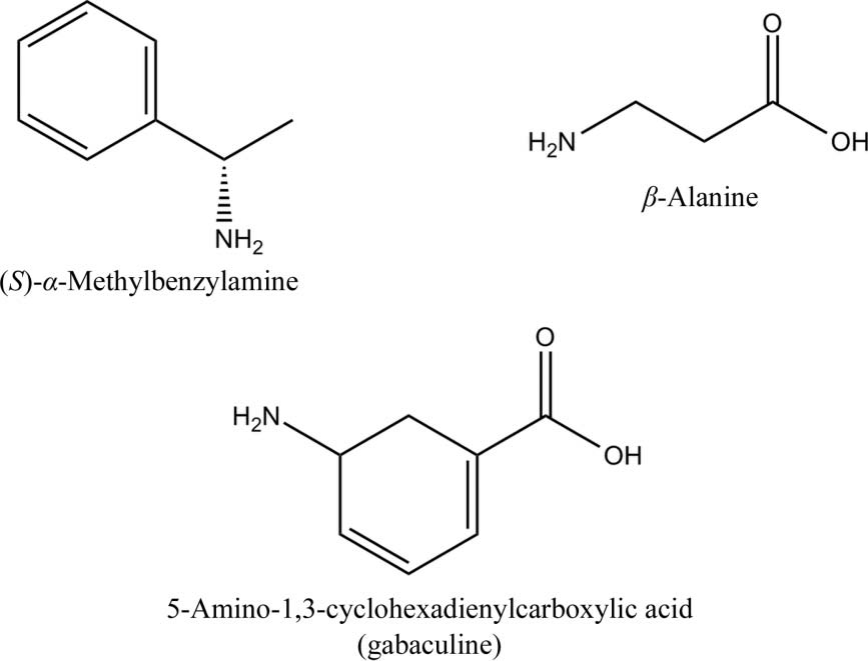
The structures of the organic compounds β-alanine, *S*-α-methylbenzyl­amine (MBA) and 5-amino-1,3-cyclohexadienylcarboxylic acid (gabaculine).

Some ATs of class III use α-ketoglutarate as an amino acceptor and some of them only accept pyruvate. The β-­alanine:pyruvate ATs (β-A:PyATs; EC 2.6.1.18) are a group of ωATs which catalyse amino transfer from β-alanine to pyruvate to produce alanine and hydroxypyruvate. There are several ωATs with high sequence similarity to β-A:PyATs which also use pyruvate as an amino acceptor and exhibit activity towards donor substrates containing no carboxyl group such as (*S*)-α-methylbenzylamine (MBA; Fig. 1 ▶). However, no activity towards β-alanine was detected for these enzymes, which were therefore named amine:pyruvate ATs (Am:PyATs; Shin *et al.*, 2003 ▶). These enzymes offer wider applications for industrial biocatalytic processes. The Am:PyAT from *Vibrio fluvalis*, which is inert to β-alanine, has been extensively studied for its use in the synthesis of chiral amines (Shin *et al.*, 2003 ▶; Yun *et al.*, 2005 ▶; Cho *et al.*, 2008 ▶). Other pyruvate-specific ωATs studied to date include those from *Klebsiella pneumonia* (Shin & Kim, 1999 ▶), *Bacillus thuringiensis* JS64 (Shin & Kim, 1999 ▶), *Pseudomonas putida* (Yonaha *et al.*, 1992 ▶), *Alcaligenes denitrificans* (Yun *et al.*, 2004 ▶), *Caulobacter crescentus* (Hwang *et al.*, 2008 ▶) and *Arthrobacter* sp. KNK168 (Iwasaki *et al.*, 2006 ▶). Recently, ωATs from *Ochrobactrum anthropi*, *Acinetobacter baumannii* and *Acetobacter pasteurianus* have also been characterized (Park *et al.*, 2012 ▶). The first crystal structure of a pyruvate-specific ωAT to be determined was that of holo ω-amino-acid:pyruvate AT from *Pseudomonas* sp. F-126 (Watanabe *et al.*, 1989 ▶).

The β-A:PyAT from *P. aeruginosa* accepts both β-alanine and MBA as amino-group donors and uses pyruvate as an amine acceptor. The enzyme is of industrial interest, as demonstrated by the synthesis of amino alcohols in a coupled reaction with *Escherichia coli* transketolase (Ingram *et al.*, 2007 ▶).

The ωAT from *Chromobacterium violaceum* (Am:PyAT) is inert towards β-alanine and uses MBA as a donor and pyruvate as an amine acceptor. It has been biochemically characterized and has been shown to have a broad substrate specificity (Kaulmann *et al.*, 2007 ▶; Schell *et al.*, 2009 ▶). We have previously reported the crystallization and preliminary crystallographic studies of this enzyme (Sayer *et al.*, 2007 ▶). The structures of the apoenzyme and holoenzyme have been reported by Humble *et al.* (2012 ▶) and by ourselves in this study. We also present the crystal structure of *C. violaceum* Am:PyAT in complex with the inhibitor gabaculine. In addition, the crystal structures of β-A:PyAT from *P. aeruginosa* in the holoenzyme and gabaculine-bound forms have been determined.

The understanding of the structural features responsible for AT substrate specificity will allow improvements for rational mutagenesis to redesign the enzyme to accept substrates for a specific industrial application. It will also allow an understanding of the enantioselectivity of the reaction and will direct mutagenesis experiments to change the AT enzyme to be either (*R*)- or (*S*)-selective. This is a significant problem with regard to the application of these enzymes as commercial biocatalysts, and *in silico* prediction of the enantiopreference of AT enzymes has been carried out using sequence alignments (Höhne *et al.*, 2010 ▶). Clearly, more structural information on different AT enzymes in complex with inhibitors or substrates will help in understanding these properties.

Gabaculine (5-amino-1,3-cyclohexadienylcarboxylic acid; Fig. 1 ▶) is a common suicide inhibitor of both α- and ω-­aminotransferases. Many different AT enzymes have been reported to be inhibited by gabaculine, including the class III ATs ornithine AT (Jung & Seiler, 1978 ▶; Shah *et al.*, 1997 ▶), *Pseudomonas* ωAT (Burnett *et al.*, 1980 ▶), 7,8-diamino­pelargonic acid synthase (Mann *et al.*, 2005 ▶) and 4-­amino­butyrate AT (Kim *et al.*, 1981 ▶). Gabaculine binds to the AT enzyme to form a Schiff base with the PLP cofactor. The complex then undergoes a number of bond rearrangements to form an unstable intermediate, which is spontaneously converted to *m*-carboxyphenylpyridoxamine phosphate (mCPP). This compound results in an irreversible aromatic modification of the cofactor, in which the Schiff base formed between gabaculine and PLP becomes a non­hydrolysable single bond (Rando, 1977 ▶; Shah *et al.*, 1997 ▶; Fu & Silverman, 1999 ▶). Despite the studies described above, no structure of an mCPP complex of an Am:PyAT or β-A:PyAT ωAT has been reported.

This paper describes the first inhibitor-bound structures of the Am:PyAT and β-A:PyAT ωAT enzymes. These results are fundamentally important to understand the mechanistic enzymology and substrate specificity of these enzymes, which together provide vital information for their exploitation as industrial biocatalysts.

## 2. Materials and methods   

### 2.1. Protein expression and purification   

The β-A:PyAT gene was cloned from *P. aeruginosa* PAO1 into the expression vector pET-24a (Novagen) and was overexpressed in *E. coli* BL21 Gold (DE3) as described by Ingram *et al.* (2007 ▶). The gene coding for *C. violaceum* Am:PyAT was cloned into the expression vector pET29a (Novagen) and was overexpressed in *E. coli* BL21 Star (DE3) pLysS (Kaulmann *et al.*, 2007 ▶). Both clones incorporated an N-terminal six-His tag for ease of protein purification and were kindly provided by Professor J. Ward (UCL, London). *E. coli* cells harbouring the pET-24a vector with the *P. aeruginosa* β-A:PyAT gene and *E. coli* BL21 Star (pLysS) cells harbouring the pET29a vector containing the *C. violaceum* Am:PyAT gene were grown in LB medium containing 30 µg ml^−1^ kanamycin at 310 K to an optical density at 600 nm of 0.8–1.0. Protein expression was induced with 1 m*M* isopropyl β-d-1-thio­galactopyranoside for 4 or 5 h at 310 K. The cells were harvested by centrifugation at 12 000*g*. The cell paste from a 2 l culture was resuspended at a concentration of 10%(*w*/*v*) in 50 m*M* Tris–HCl pH 7.5. Sonication was carried out using a Soniprep 150 sonicator (Sanyo) followed by centrifugation at 12 000*g* to remove precipitated protein and cell debris. The aminotransferases were purified on a HiLoad nickel column (Pharmacia, Uppsala, Sweden) using a linear gradient of 0–1 *M* imidazole in a buffer consisting of 50 m*M* Tris–HCl pH 7.5, 50 µ*M* PLP. The enzymes were further purified by gel filtration on a Superdex 200 gel-filtration column (Pharmacia, Uppsala, Sweden) using a buffer consisting of 50 m*M* Tris–HCl pH 7.5, 0.1 *M* NaCl, 50 µ*M* PLP. Dynamic light scattering was measured using a DynaPro Titan instrument (Wyatt Technology, Santa Barbara, USA) at 292 K.

### 2.2. Crystallization and data collection   

*C. violaceum* Am:PyAT was crystallized by the microbatch method using an Oryx Robot (Douglas Instruments) with commercial crystal screens from Molecular Dimensions. 1 µl protein sample (10 mg ml^−1^) was mixed with an equal volume of reservoir solution. For initial crystallization, 100 µ*M* PLP was added to the protein solution prior to concentration (sample *A*). The first *C. violaceum* Am:PyAT crystals grown from sample *A* were obtained using 0.1 *M* lithium sulfate monohydrate, 50 m*M* Tris–HCl pH 8.5, 15%(*w*/*v*) PEG 4000. However, these crystals did not contain bound PLP and the resulting structure was that of the apoenzyme (Sayer *et al.*, 2007 ▶). In order to obtain crystals of the holoenzyme, PLP was added to the concentrated protein sample to a final concentration of 10 m*M* (sample *B*). To obtain crystals of the inhibitor complex, 6 m*M* gabaculine was added to the protein sample in addition to 10 m*M* PLP (sample *C*). These samples (*B* and *C*) both crystallized using 0.05 *M* HEPES pH 7.5, 5%(*v*/*v*) 2-propanol, 10%(*w*/*v*) PEG 4000. The apoenzyme crystals of *C. violaceum* Am:PyAT were cooled straight from the droplet and data were collected in-house as described by Sayer *et al.* (2007 ▶). Crystals grown from samples *B* and *C* were cooled under silicon oil and data were collected at 100 K using an ADSC detector on beamlines 10.1 and 14.1 of the Daresbury Synchrotron, England, respectively. Data were processed using the programs *DENZO* and *SCALEPACK* (Otwinowski & Minor, 1997 ▶), *MOSFLM* (Leslie & Powell, 2007 ▶) and *SCALA* (Evans, 2006 ▶). The space group of the apoenzyme crystals was *P*1 and the unit-cell parameters were *a* = 58.9, *b* = 61.9, *c* = 63.9 Å, α = 71.9, β = 87.0, γ = 74.6°. The unit cell contained a dimeric ωAT molecule, giving a solvent content of 40.4% and a *V*_M_ of 2.1 Å^3^ Da^−1^. The inhibitor complex (sample *C*) crystallized in the same space group with similar unit-cell parameters. The holoenzyme crystals (sample *B*) had a triclinic unit cell with similar *b* and *c* unit-cell parameters; however, the *a* unit-cell parameter was approximately double that of the crystals of the apoenzyme. These crystals contained two dimeric molecules of *C. violaceum* Am:PyAT in the unit cell. As all crystals of *C. violaceum* Am:PyAT crystallized in space group *P*1, the completeness of the data was in the range 88–94%. This arises from the inability to collect reflections close to the rotation axis when using the rotation method (Arndt & Wonacott, 1977 ▶) and the absence of their symmetry equivalents not close to the axis in the triclinic space group (Table 1 ▶).

The *P. aeruginosa* β-A:PyAT protein was crystallized using the microbatch method. 1 µl protein solution (10 mg ml^−1^ containing 50 µ*M* PLP) was mixed with an equal volume of reservoir solution consisting of 50 m*M* Tris–HCl pH 8.5, 20% PEG 200. Inhibitor-complex crystals were grown in the presence of 1 m*M* gabaculine and 1 m*M* PLP using a reservoir solution consisting of 2.4 *M* unbuffered sodium malonate. Crystals of both the holoenzyme and the inhibitor complex were cooled directly from the droplet as both crystallization conditions do not produce significant ice rings when frozen. Diffraction data were collected at 100 K on beamline I03 at the Diamond Synchrotron, England. The holoenzyme data were collected on an ADSC detector and were processed using *XDS* (Kabsch, 2010 ▶) through the *xia*2 pipeline (Winter, 2010 ▶). The data were indexed in space group *P*2_1_, with unit-cell parameters *a* = 80.4, *b* = 133.2, *c* = 162.0 Å, β = 92°. There were eight monomers in the asymmetric unit, giving a *V*_M_ of 2.2 Å^3^ Da^−1^; 45% of the crystal volume was occupied by solvent. The inhibitor-complex data were collected using a PILATUS 6M detector and processed using *XDS* in the *xia*2 pipeline. The inhibitor-complex space group was determined as orthorhombic *P*2_1_2_1_2_1_, with unit-cell parameters *a* = 119.2, *b* = 192.5, *c* = 77.3 Å. All of the crystallographic axes were assigned as screw axes on the basis of the observed systematic absences. The asymmetric unit contained four monomers with 46% solvent content, giving a *V*_M_ of 2.3 Å^3^ Da^−1^.

### 2.3. Structure solution and refinement   

The *C. violaceum* Am:PyAT apoenzyme structure was originally solved by molecular replacement (MR) using chain *A* of 7,8-diaminopelargonic acid synthase as a model (PDB entry 1qj3; Käck *et al.*, 1999 ▶) as described in Sayer *et al.* (2007 ▶). The holoenzyme structure of *C. violaceum* Am:PyAT and the structure of its complex with gabaculine were solved by MR with *MOLREP* (Vagin & Teplyakov, 2010 ▶) using the refined apoenzyme structure as a model.

The structure of holo *P. aeruginosa* β-A:PyAT was solved by MR with *MOLREP* using the structure of holo *C. violaceum* Am:PyAT as a model. The structure of the inhibitor complex of *P. aeruginosa* β-A:PyAT was solved by MR with *MOLREP* using the refined holo *P. aeruginosa* β-­A:PyAT structure as a model. The solution and refinement of both the holoenzyme structure and the inhibitor complex of *P. aeruginosa* β-­A:PyAT encountered ambiguities in either the origin or space-group assignment, as discussed in §[Sec sec3.1]3.1.

The resulting models of the *C. violaceum* and *P. aeruginosa* structures underwent cycles of crystallographic refinement using *REFMAC*5 (Murshudov *et al.*, 2011 ▶), and manual model building was performed in *Coot* (Emsley *et al.*, 2010 ▶). The ligand dictionaries for refinement were prepared using *JLigand* (Lebedev *et al.*, 2012 ▶). Solvent molecules were added using *Coot*.

The atomic coordinates and structure factors for *P. aeruginosa* β-A:PyAT in holo and gabaculine-bound forms have been deposited in the PDB as entries 4b9b and 4b98, respectively. The atomic coordinates and structure factors for *C. violaceum* Am:PyAT in apo, holo and gabaculine-bound forms have been deposited in the PDB as entries 4ba4, 4ah3, 4ba5, respectively.

## 3. Results and discussion   

### 3.1. Pseudosymmetry problems in structure solution and refinement   

The native Patterson synthesis of holo *P. aeruginosa* β-­A:PyAT calculated at 3 Å resolution contained a strong pseudo-translation peak with a height of 35% of the origin peak at (0, 0, 0.5). This peak indicates the presence of pairs of molecules related by pseudo-translation. The cross-rotation function was calculated with an integration radius of 30 Å in the resolution range 15–3 Å using a dimeric model of holo *C. violaceum* Am:PyAT. There were four strong cross-rotation peaks (the heights of the correct peaks were 7.2–7.4σ, with a background of 4.8σ). The translation function was calculated at 15–4.5 Å resolution using *MOLREP*. Four dimeric molecules (eight monomers) were positioned with a final correlation coefficient of 0.419. The solution contained two pairs of dimers with close orientations in each pair, in agreement with the presence of translational pseudosymmetry. This solution was found by switching off the pseudotranslation search option in *MOLREP* using a single dimer as a search model at each stage of the search. This was carried out because the default search for pairs of models related by pseudotranslation did not give high-contrast solutions for orientations that were thought to be correct.

The rigid-body refinement of the MR solution at 15–4 Å resolution was followed by restrained refinement with isotropic *B* factors using *REFMAC*5. The phases obtained by eightfold NCS averaging using the program *DM* (Cowtan, 2010 ▶) were further used for phased refinement in *REFMAC*5 and the model was extensively rebuilt using *Coot*. However, an *R* factor of 0.44 and an *R*_free_ of 0.49 at 1.8 Å resolution were the best refinement statistics that could be achieved, suggesting that MR could have resulted in a false origin solution or, in other words, the pseudosymmetry axes could have been misinterpreted as symmetry axes by the MR program (Isupov & Lebedev, 2008 ▶; Lebedev & Isupov, 2012 ▶). To correct this false solution, two actions were carried out: (i) an asymmetric unit was selected that included two pairs of dimers related by pseudotranslation (not by pseudosymmetry rotations) and (ii) this asymmetric unit was translated by **c**/4. The corrected structure was refined to a *R* factor of 0.39 and an *R*_free_ of 0.44 before any manual rebuilding.

The structure of the inhibitor complex of *P. aeruginosa* β-­A:PyAT was solved by MR with *MOLREP* using the refined *P. aeruginosa* β-A:PyAT holoenzyme as a model. The space group of the inhibitor complex was assigned by *xia*2 as *P*2_1_2_1_2_1_ from systematic absences, with unit-cell parameters *a* = 119.2, *b* = 192.5, *c* = 77.3 Å. The native Patterson synthesis calculated at 3 Å resolution contained a strong pseudotranslation peak at (0, 0.5, 0.5) with a height of 71% of the origin Patterson peak. While there were no reasons to doubt the twofold crystallo­graphic screw axis along **a**, any of the axes along **b** and **c** could have been either a proper or a screw rotational twofold axis, since the observed systematic absences could have been caused by pseudotranslation.

Given that the point group of the crystal is 222, the pseudotranslation (**b** + **c**)/2 and symmetry axes along **b** and **c** generate pseudosymmetry axes along **b** and **c**, respectively. However, because the pseudotranslation is a diagonal translation, the generated axes are screw axes if the crystallographic axes are proper axes and *vice versa*. Therefore, the problem of distinguishing the true structure from false structures in which the crystallographic axes are misinterpreted as pseudosymmetry axes is now reduced to a choice of one of the space groups *P*2_1_22, *P*2_1_22_1_, *P*2_1_2_1_2 and *P*2_1_2_1_2_1_. This consideration also suggests that one could expect convincing MR solutions for all of the space groups in this set.

Indeed, the translational search in these four space groups resulted in high-contrast solutions in which the two top correlation coefficients were almost identical at 0.574 and 0.572. These were obtained in space groups *P*2_1_2_1_2 and *P*2_1_2_1_2_1_, respectively. Subsequent refinement favoured the second space group. After 60 cycles of restrained refinement with *REFMAC* at 1.65 Å resolution, the *R*_free_ converged to 0.394 for the *P*2_1_2_1_2 structure and to 0.311 for the *P*2_1_2_1_2_1_ structure.

In addition, because the Patterson peak at (0, 0.5, 0.5) was so strong, we could not completely exclude the possibility that this peak corresponded to the true crystallographic translation and that the space group was actually *A*2_1_22 (in the crystal setting under consideration with *a* = 119.2, *b* = 192.5, *c* = 77.3 Å) and that half of the measured reflections were merely noise. The program *REINDEX* from the *CCP*4 program suite (Winn *et al.*, 2011 ▶) was used to change the crystal setting to the conventional one (*a* = 77.3, *b* = 192.5, *c* = 119.2 Å; Hermann–Mauguin symbol *C*222_1_) and to exclude reflections with *h* + *k* = 2*n* + 1. The MR solution (two monomers) found in this space group could be refined to an *R*_free_ of 0.343 at 1.65 Å resolution. As the best refinement results were previously achieved in *P*2_1_2_1_2_1_, subsequent model refinement and rebuilding was carried out in this space group using all measured reflections.

### 3.2. Quality of the models   

The three *C. violaceum* Am:PyAT and the two *P. aeruginosa* β-­A:PyAT structures were refined to a resolution equivalent to or higher than 1.7 Å. For each model the final round of refinement resulted in acceptable values of the *R* factor and *R*_free_ (Table 1 ▶). Some residues were excluded from the model when poor electron density was observed at the N- and C-­termini. The *G*-factors calculated for each model confirmed that the structures have normal stereochemical properties. The Ramachandran plots (Ramakrishnan & Ramachandran, 1965 ▶) of the models revealed that at least 88% of the residues lie in the most favoured regions. Cofactor and inhibitor molecules were positioned in the active site using *F*_o_ − *F*_c_ OMIT maps and the occupancies of these molecules or their components were assigned so that after refinement their *B* factors were consistent with those of neighbouring residues. The final refinement statistics and validation results for all of the structures are shown in Table 1 ▶. As observed previously in many other PLP-dependent enzymes, the catalytic Lys288 is amongst the Ramachandran plot outliers in all of the subunits of both the holoenzyme and the mCPP-complex structures of *P. aeruginosa* β-A:PyAT and of the holoenzyme structure of *C. violaceum* Am:PyAT. However, it is not an outlier in the apoenzyme and mCPP-complex structures of *C. violaceum* Am:PyAT. Other Ramachandran plot outliers are well defined in the electron density and are consistent between different subunits of the same structure. Pro176 of *C. violaceum* Am:PyAT is in a *cis* conformation in every subunit of all of the structures. The *P. aeruginosa* β-A:PyAT structures do not contain any residues in a *cis* conformation. Many residues were modelled with alternative conformations of their side chains. The main-chain O atoms of some residues were modelled in an alternative conformation. The most significant main-chain split was modelled for residues Ala57–Cys61 in subunit *A* of the *C. violaceum* Am:PyAT–mCPP complex structure.

### 3.3. Quaternary structure   

*C. violaceum* Am:PyAT elutes with an apparent molecular mass of 100 kDa on a size-exclusion chromatography column, which corresponds to a dimer. This enzyme is found to be a dimer in the crystal, with the asymmetric unit containing one (apoenzyme and mCPP complex) or two (holoenzyme) dimers. Formation of the *C. violaceum* Am:PyAT holoenzyme dimer buries 5600 Å^2^, which equates to 28% of the solvent-accessible area of each subunit.

**Figure 2:**
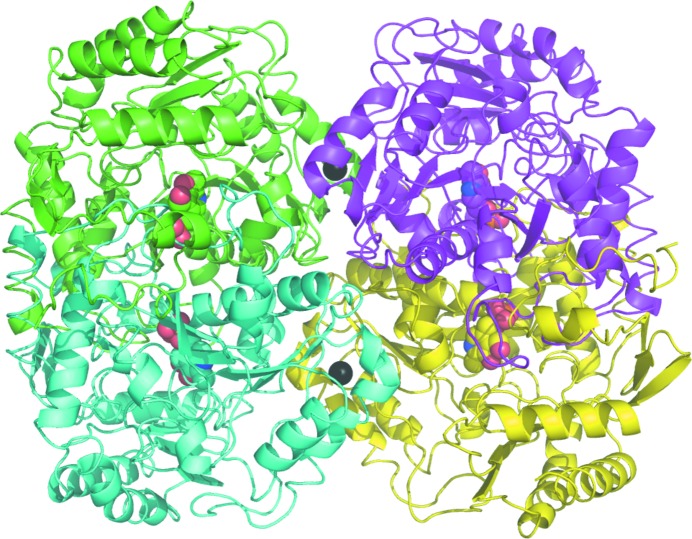
A ribbon diagram of the *P. aeruginosa* β-A:PyAT tetramer viewed approximately along the molecular dyad. The individual subunits are shown in different colours. The cofactor PLP is shown as a space-filling model and the two calcium ions on the interface of the catalytic dimers are shown as black spheres. Figs. 2–7 were prepared using *PyMOL* (DeLano, 2002 ▶).

The *P. aeruginosa* β-A:PyAT enzyme elutes from the size-exclusion chromatography column earlier than *C. violaceum* Am:PyAT, with an apparent molecular weight of 200 kDa, which indicates that it is a tetramer in solution. Dynamic light-scattering experiments estimate the approximate molecular weight of the *P. aeruginosa* β-A:PyAT species in solution to be double the size of the *C. violaceum* enzyme dimer. The asymmetric unit of the *P. aeruginosa* β-A:PyAT holoenzyme crystal structure contains two tetramers, and a single tetramer makes up an asymmetric unit in the complex structure (Fig. 2 ▶). Upon the formation of a catalytic dimer of *P. aeruginosa* β-­A:PyAT, 6340 Å^2^ (31%) of the solvent-accessible area of each subunit is buried. The interface between the catalytic dimers in the tetramer is significantly less extensive, burying 1200 Å^2^ (6%) of the solvent-accessible area of each subunit, and is filled with water molecules. Two calcium ions which probably contribute to the stability of the tetramer are located on the *P. aeruginosa* β-A:PyAT dimer–dimer interface; each is coordinated by the carboxyl groups of Asp180 of the two adjacent subunits related by a molecular dyad and by four water molecules. The calcium ions have full occupancy in both the holoenzyme and the mCPP-complex structures, although no divalent cations were intentionally added to the crystallization media.

An ω-amino-acid:pyruvate AT from *Pseudomonas* sp. F-126 which shares 78% sequence identity with *P. aeruginosa* β-­A:PyAT has also been reported to be a tetramer by both size-exclusion chromatography and analytical ultracentri­fugation (Yonaha *et al.*, 1977 ▶). The tetramers of *Pseudomonas* sp. F-126 ω-amino-acid:pyruvate AT and *P. aeruginosa* β-­A:PyAT are almost identical and bury the same amount of surface area upon formation. However, no divalent cations were located in the crystal structure of *Pseudomonas* sp. F-126 ω-amino-acid:pyruvate AT, although Asp180 is conserved.

Amongst the class III ATs with known structure, most are dimers and those that are tetramers have the same subunit arrangement as the tetramer of *P. aeruginosa* β-A:PyAT. There is a similarity between the tetramers of β-­A:PyAT and of a dialkylglycine decarboxylase from *Burkholderia cepacia* (Toney *et al.*, 1993 ▶). The latter protein catalyses a different type of reaction and has 25% sequence identity to *P. aeruginosa* β-A:PyAT. Such similarity of the tetramers is unlikely to be a coincidence and hence an evolutional relationship between the two proteins can be inferred. The tetramers of class III ATs are not similar to the tetrameric PLP-dependent lyases such as tryptophanase (Isupov *et al.*, 1998 ▶), which form their dimer–dimer interface on the opposite side of a catalytic dimer. These ωATs tetramers can be considered to be arranged in an ‘inside-out’ fashion with respect to the PLP-dependent lyases.

### 3.4. Overall fold   

**Figure 3:**
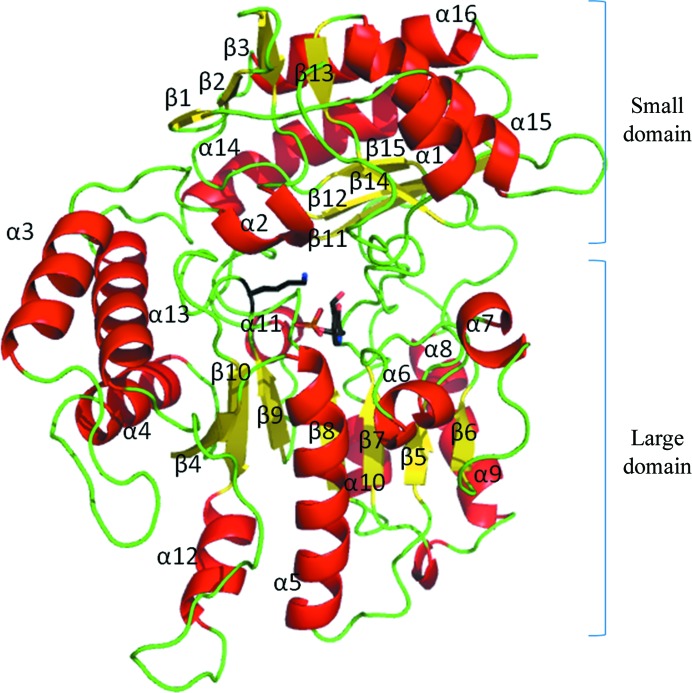
Folding of the *P. aeruginosa* β-A:PyAT subunit shown as a ribbon diagram; α-helices are shown in red, β-strands in yellow and loops in green. The secondary-structure elements are labelled. PLP and Lys288 are shown as stick models.

The *C. violaceum* Am:PyAT and *P. aeruginosa* β-A:PyAT enzymes are similar to other class III ATs and are folded into two α/β domains (Fig. 3 ▶). The topology of this arrangement corresponds to the domains of the general PLP-dependent fold I enzymes (Schneider *et al.*, 2000 ▶), consisting of a large and a small domain, with the latter comprising the N- and C-­terminal parts of the polypeptide chain.

The large domain folds into a typical α/β/α sandwich made up of a central seven-stranded β-sheet with topology +5x, +1x, −2x, −1x, −1x, −1 (Richardson, 1981 ▶) and direction + − + + + + +. The small domain, which is larger in ωATs in comparison with most αATs, is made up of two β-sheets. The four-stranded N-terminal sheet is of mixed type with direction + − + + and topology +1, +1, +1x, with the last β-strand coming from the C-­terminal part of the domain. The C-­terminus of the small domain is built around an antiparallel β-sheet with topology +1, +2x, −1 which is shielded from solvent by three α-helices on one side. The other side of this sheet faces the large domain and forms a crevice between the two domains to accommodate the active site.

Significant structural rearrangements accompany cofactor binding in *C. violaceum* (CV) Am:PyAT, as also described by Humble *et al.* (2012 ▶). This includes unwinding of the α-helix (residues CV 317–322) to form a loop covering the PLP phosphate group. This movement is ‘hinged’ on residues CV Gly313 and CV Gly324. This unwound loop conformation is normally observed in both the apo and the holo structures of other class III ATs. The N-terminus (up to residue 36), which is disordered in the apoenzyme structure, becomes ordered in the holoenzyme structure and occupies the position of the unwound helix.

### 3.5. The unusual cofactor binding   

Most PLP enzymes have high affinity for the cofactor, which is normally found at high occupancy in the active site of the enzyme, forming a Schiff base (an internal aldimine) with the active-site lysine. However, this was not the case for either of the ωATs considered here.

Attempts to crystallize *C. violaceum* Am:PyAT with a small excess of PLP (100 µ*M*) resulted in the structure of the apoenzyme (Sayer *et al.*, 2007 ▶). Crystallization with a significant excess of cofactor (at least 5 m*M*) was required to obtain holoenzyme crystals at pH 8.5. Interestingly, Humble *et al.* (2012 ▶) obtained crystals of the *C. violaceum* Am:PyAT holoenzyme using a different approach. Instead of increasing the concentration of PLP, they cocrystallized the enzyme in the presence of 50 µ*M* PLP, 1 m*M* of the acceptor substrate pyruvate and 1 m*M* of the poor donor substrate isopropyl­amine, which enforced a high occupancy of PLP in the active site. The holoenzyme structure presented here has full occupancy of the cofactor in the active site, which forms the internal aldimine link to the active-site Lys288. The low affinity of the enzyme for the cofactor could be a mechanism of regulation of activity *in vivo*. It has been reported that owing to its low affinity for the cofactor the *C. violaceum* Am:PyAT enzyme can be used industrially for the synthesis of PMP (Schell *et al.*, 2009 ▶).

Our understanding is that unlike other PLP enzymes, the cofactor binding in *C. violaceum* Am:PyAT does not significantly reduce the free energy of the system. This may be owing to the fact that the large structural rearrangements that occur upon cofactor binding result in the energetically unfavourable breakdown of many hydrogen bonds.

The *P. aeruginosa* β-A:PyAT holoenzyme has high occupancy of the cofactor in the active site when crystallized with a small excess of PLP. However, according to the electron density observed, the dominant species of the cofactor in the active site is free PLP. Originally, we assumed that the crystals contained a PMP complex with an amino group acquired from an unknown donor substrate during purification. However, incubation and cocrystallization of the protein with 20 m*M* of the acceptor substrate pyruvate, which should have restored the internal aldimine form of the cofactor, resulted in a structure with the same electron density for free PLP in the active site (data not shown).

The occupancy refinement of the *P. aeruginosa* β-A:PyAT holoenzyme structure was performed using *REFMAC* v.5.7 with the two species, Schiff-base PLP–Lys288 and free PLP, present simultaneously in the active site. The resulting overall occupancy of the cofactor varied in the range 0.70–0.78 in the eight different subunits. The internal aldimine species refined to occupancies in the range 0.07–0.20, with the ratio of the occupancy of internal aldimine species to the overall occupancy of the cofactor varying in the range 0.10–0.28. The low occupancy of the internal aldimine species agrees with the lack of continuous 2*F*_o_ − *F*_c_ electron density for the Schiff base in the active site at a 1σ cutoff. The internal aldimine species were not included in the refined model of the *P. aeruginosa* β-­A:PyAT holoenzyme owing to the low occupancy.

In the structure of a related ω-amino acid:pyruvate AT from *Pseudomonas* sp. F-126 (Watanabe *et al.*, 1989 ▶) the cofactor was also modelled as free PLP, although the electron density suggested that some proportion of the active-site species was in the internal aldimine form. This is consistent with earlier spectroscopic studies on this protein (Yonaha *et al.*, 1983 ▶), in which only one mole of PLP per mole of tetrameric protein was observed to form an internal aldimine when monitored using the absorption spectrum at 400 nm, which is equivalent to 25% occupancy. It would appear that while the PLP cofactor binds to the *P. aeruginosa* β-A:PyAT enzyme very tightly, formation of the external aldimine requires a small conformational change away from the minimal energy position, making free PLP bound in the enzyme active site the most energetically favourable species.

### 3.6. PLP-binding site   

In both ωATs PLP binds between the two domains of a single subunit at the interface of the two subunits in the catalytic dimer. Residues from both subunits are involved in cofactor binding, but the active-site cleft is mainly made up of residues from one subunit. The cofactor is bound at the bottom of the active site, with its *re* side facing the solvent. The active-site Lys288 is located between β-strands 9 and 10 and is located on the *si* face of the cofactor which is shielding the lysine from the solvent. In *P. aeruginosa* (PA) β-A:PyAT the phosphate group of PLP makes hydrogen bonds to the main-chain amides of PA Gly120 and PA Thr327 and the side chains of PA Thr327 and PA Ser121. The carboxyl group of PA Asp259 makes a hydrogen bond to the pyridine-ring N atom of PLP. PA Asp259 is kept in place by interactions with the imidazole ring of PA His154. The pyridine ring of PLP is sandwiched between the side chains of PA Tyr153, which lies perpendicular to the cofactor ring on the *re* side, and PA Val261 on the *si* side of the ring.

**Figure 4:**
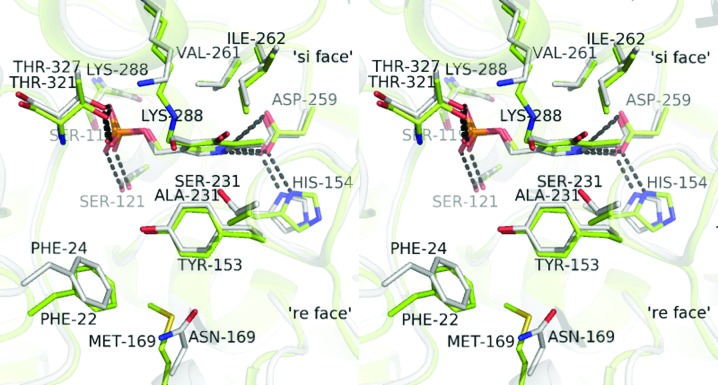
A stereo representation of the active sites of the holoenzyme structures of *C. violaceum* Am:PyAT (green) and *P. aeruginosa* β-A:PyAT (grey) shown superimposed. The cofactor molecules and side chains of the residues within 4.5 Å of the cofactor are shown as stick models. The active-site Lys288 forms a Schiff base with the cofactor PLP in *C. violaceum* Am:PyAT. In *P. aeruginosa* β-A:PyAT the cofactor is modelled as free PLP. Cofactor molecules and neighbouring residues are shown as stick models.

The holo structures of the two enzymes are similar in conformation (Fig. 4 ▶) and both are similar to the structures of other holo­enzymes of class III ATs which are available in the PDB.

### 3.7. Gabaculine cocrystallization   

The *P. aeruginosa* enzyme has a narrow amine-substrate specificity for β-alanine, 4-­aminobutyrate and MBA in the presence of pyruvate. Its very close homologue *Pseudomonas* sp. F-126 ω-amino-acid:pyruvate AT shows high activity towards ω-­amino acids and alanine, very limited activity towards glycine and no detectable activity towards most other α-amino acids (Yonaha *et al.*, 1977 ▶). The *C. violaceum* enzyme shows no activity towards β-alanine, but has relatively broad substrate specificity for aromatic and aliphatic amines and activity towards amino alcohols and some α-­amino acids (Kaulmann *et al.*, 2007 ▶).

We were unable to explain the wealth of substrate-specificity data and the differences between the two enzymes from knowledge of just the holoenzyme structures and the apoenzyme structure of *C. violaceum* Am:PyAT. The structures of the two holo­enzymes were similar. Many unsuccessful cocrystallization experiments were carried out using different substrates and substrate analogues of the two enzymes in order to trap an intermediate complex. Only cocrystallization with gabaculine allowed us to obtain structures of an inhibitor complex for the two enzymes. The cocrystallization experiments with *C. violaceum* Am:PyAT required high concentrations of both cofactor and inhibitor. The inhibitor-bound complexes of both enzymes have provided important information regarding the active-site cavities of the different enzymes which has allowed an interpretation of their observed substrate specificity.

Inhibition studies have previously shown that gabaculine fully inhibits *C. violaceum* Am:PyAT in the presence of both amine donor and acceptor substrates. However, free PMP formation is observed with pre-incubated gabaculine-bound enzyme in the presence of the amine donor MBA and excess PLP (Schell *et al.*, 2009 ▶). We attribute this to a lower affinity of mCPP for the active site of the *C. violaceum* Am:PyAT enzyme, which is a consequence of the low affinity of this enzyme for PLP. Owing to the lower affinity for mCPP this can be replaced by PLP in the active site, which can then be transaminated to PMP.

### 3.8. Gabaculine complex   

**Figure 5:**
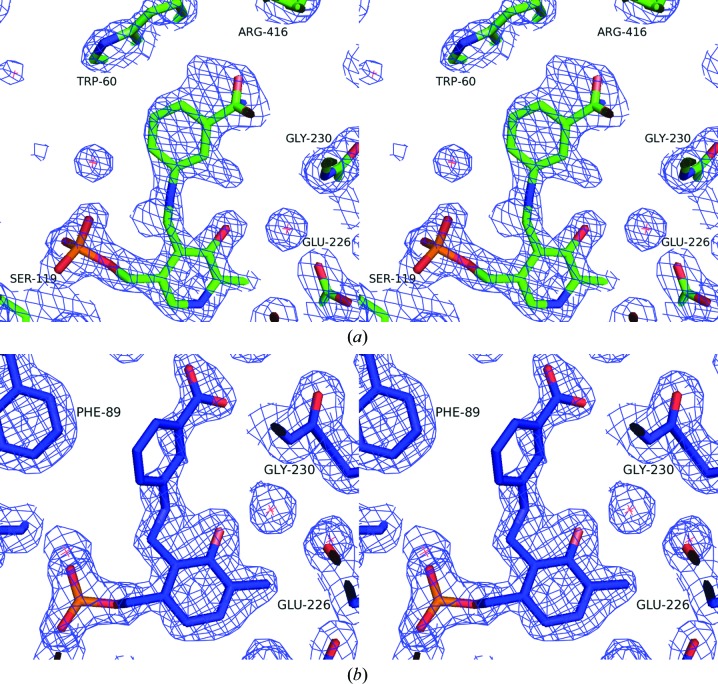
A stereo representation of the 2*F*_o_ − *F*_c_ electron-density maps, contoured at 1σ, for the active sites of the gabaculine complexes of *C. violaceum* Am:PyAT (*a*) and *P. aeruginosa* β-A:PyAT (*b*). The mCPP molecule and neighbouring residues are shown as stick models.

The electron-density maps clearly show gabaculine covalently bound to C4′ of PLP as the mCPP complex in four chains of the *P. aeruginosa* enzyme and in both chains of the *C. violaceum* enzyme (Fig. 5 ▶). Refinement of the occupancy of the two conformations of mCPP in the active sites of the *P. aeruginosa* β-A:PyAT complex using *REFMAC*5 resulted in occupancies of mCPP of close to 1 in all four subunits. The *C. violaceum* Am:PyAT–mCPP complex was refined with partial occupancies of 0.7 and 0.6 in subunits *A* and *B*, respectively. The loop CV Ala57–Cys61 in chain *A* of the *C. violaceum* Am:PyAT–mCPP complex was built in two alternative conformations. The minor conformation of this loop in chain *A* with 0.3 occupancy is the same as the single conformation in subunit *B*. This is similar to the conformation observed in the holoenzyme structure of the *C. violaceum* AT. The major conformation of this loop in chain *A* (occupancy of 0.7) folds differently, with the positions of CV Leu59 and CV Trp60 inverted at the bottom of the substrate pocket. The mCPP aromatic ring is rotated by approximately 15° between the two subunits of the *C. violaceum* Am:PyAT–mCPP complex.

### 3.9. Conformational changes in the *C. violaceum* mCPP complex   

**Figure 6:**
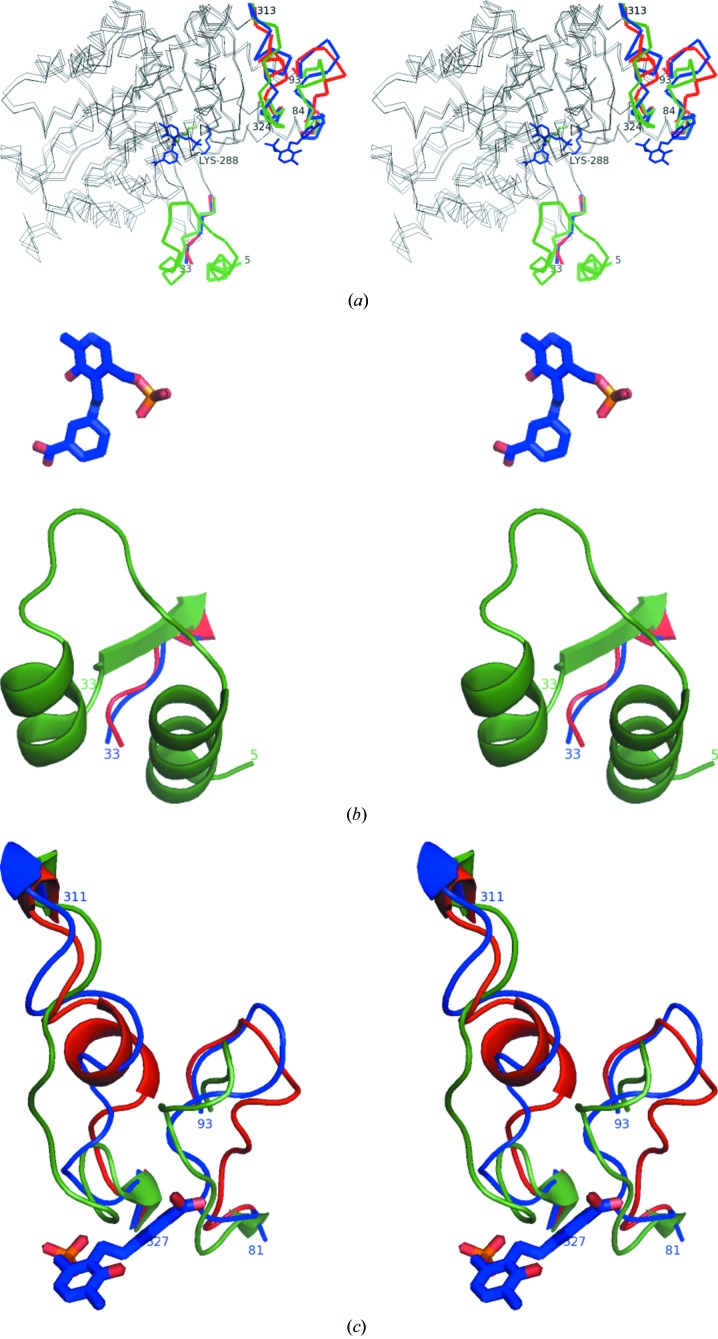
Stereo representations of the conformational changes between the different forms of *C. violaceum* Am:PyAT. (*a*) Superposition of the C^α^ traces of the apoenzyme (red), the holoenzyme (green) and the gabaculine complex (blue). The relatively stationary parts of the protein are shown in grey. (*b*) The conformational changes of the N-terminal region displayed as a cartoon with the same colour scheme as in (*a*). (*c*) The conformational changes of the loop regions 81–93 and 311–327 displayed as a cartoon with the same colour scheme as in (*a*).

Surprisingly, the two enzymes undergo very different structural rearrangements between the holoenzyme structure and the mCPP complex. In the *C. violaceum* Am:PyAT enzyme the movements of loops upon inhibitor binding are as extensive as those between the apo and holoenzyme structures (Fig. 6 ▶). In the *P. aeruginosa* enzyme there is almost no movement, with all residues in the active site retaining a permanent position. Therefore, *P. aeruginosa* β-A:PyAT can be described as having a more rigid scaffold than *C. violaceum* Am:PyAT.

**Figure 7:**
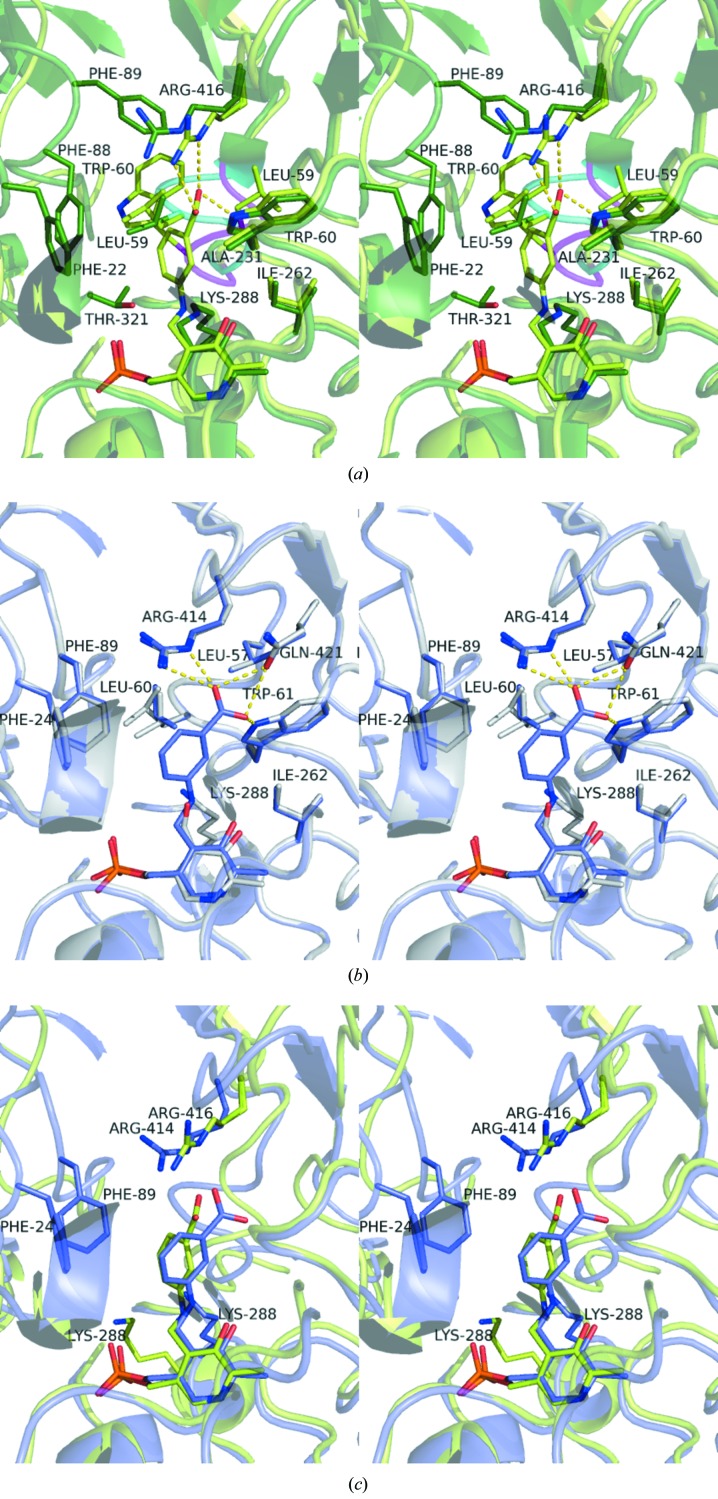
Stereo representation comparing the gabaculine complexes of the *C. violaceum* Am:PyAT and *P. aeruginosa* β-A:PyAT enzymes. The side chains of residues within 4.5 Å of the mCPP inhibitor are shown as stick models. (*a*) The interactions of the mCPP bound in the *C. violaceum* Am:PyAT active site (light green). The residues of the holoenzyme are superimposed (dark green), highlighting the movements associated with inhibitor binding to the active site. The differences in the conformations of the Ala57–Cys61 loop are shown in magenta for the gabaculine-bound structure and in cyan for the holoenzyme structure. (*b*) The structure of mCPP-bound *P. aeruginosa* β-A:PyAT (blue) superimposed on the structure of its holoenzyme (grey). (*c*) The superposition of the active sites of the mCPP-complex structures of *C. violaceum* Am:PyAT (green) and *P. aeruginosa* β-A:PyAT (blue), highlighting the different orientations of mCPP observed between the two enzymes.

The N-terminal region (residues 5–32) was built in the *C. violaceum* Am:PyAT holoenzyme structure (Figs. 6 ▶ *a* and 6 ▶ *b*); however, it was not possible to build it in the apoenzyme and the mCPP complex owing to the absence of continuous electron density. The position of the N-terminal region in the holoenzyme structure occupies the same space as the flexible loop region 313–324 (Fig. 6 ▶ *b*) in the mCPP complex and apoenzyme structures, confirming the displacement of the N-­terminus. Residue CV Thr321, which is conserved among ATs, forms hydrogen bonds to the phosphate of the PLP through peptide and side-chain interactions in the holo­enzyme. This is displaced away from the active-site region in the mCPP-complex and apoenzyme structures, adopting a helical configuration. Two glycine residues, CV Gly313 and CV Gly324, act as flexible hinges at either end of this loop. The position of this loop region in the mCPP-bound enzyme is ‘halfway’ between the apoenzyme and holoenzyme structures. The N-terminal region of the holoenzyme projects residue CV Phe22 into the active site, affecting the substrate specificity of the enzyme (Fig. 7 ▶ *a*).

The loop region CV 84–93 (Figs. 6 ▶ *a* and 6 ▶ *c*) is also observed in a conformation closer to the cofactor in the holoenzyme structure compared with the apoenzyme and gabaculine-bound structures. In the holoenzyme CV Phe88 and CV Phe89 are positioned in the active site, while in the apoenzyme and gabaculine-bound structures the two residues point towards the exterior of the protein (Fig. 7 ▶ *a*). The active sites of the *P. aeruginosa* and *C. violaceum* AT enzymes acquire different conformations upon mCPP binding. A comparison of the flexible CV Gly313–Gly324 region with the corresponding PA region Asn312–Ala330 reveals that the glycine residues allowing flexibility in the *C. violaceum* ωAT structure are absent in the *P. aeruginosa* enzyme. The larger extended loop region of *P. aeruginosa* β-A:PyAT forms additional inter­actions with the neighbouring helical regions PA 24–29 and PA 394–400 which are expected to make the loop less flexible than that found in the equivalent *C. violaceum* Am:PyAT region. The flexibility of the *C. violaceum* Am:PyAT scaffold is further demonstrated by significant changes in the orientation of the active-site lysine and rearrangement of the CV Ala57–Cys61 loop in the substrate-binding pocket on mCPP-complex formation (Fig. 7 ▶ *a*).

The observed changes in the conformation of *C. violaceum* Am:PyAT do not agree with the proposal by Humble *et al.* (2012 ▶) that the binding of the phosphate group of the cofactor is the driving force behind the significant conformational changes between the apoenzyme and the holoenzyme. The mCPP complex with the phosphate group bound in the same place is almost as open as the apoenzyme structure. The prediction by the same authors that the binding of substrate will make the active site even more closed is not supported by the structure of the mCPP complex reported here.

### 3.10. Substrate-binding site of *P. aeruginosa* β-A:PyAT   

**Figure 8:**
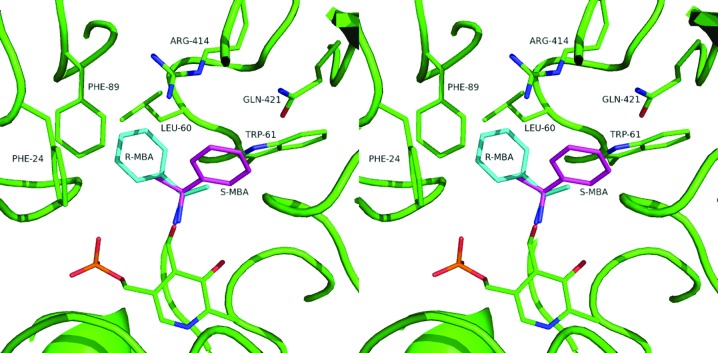
Stereo representation showing the enantioselectivity towards the *S*-MBA substrate in the *P. aeruginosa* β-A:PyAT active site based on the gabaculine complex and the requirement for the scissile C^α^—H bond to be normal to the pyridine ring of PLP. The modelled *R*-MBA clashes with the neighbouring residues PA Leu60 and PA Phe89.

The inhibitor is bound on the *re* face of the cofactor at the bottom of the active site, although it binds differently in the two enzymes. In *P. aeruginosa* β-A:PyAT the carboxyl group of mCPP makes hydrogen bonds to the side-chain O atom of PA Gln421 and the side-chain N atoms of PA Trp61 and PA Arg414 (Fig. 7 ▶ *b*). These three residues form a rigid substrate carboxyl group-binding site. When β-alanine is modelled into the structure with the carboxyl group bound to this site, its amino group is ideally positioned for formation of the external aldimine and the subsequent transamination reaction. This is further favoured by the conserved position of PA Lys288, which is involved in proton abstraction. The structure of the active site of ω-amino acid:pyruvate AT from *Pseudomonas* sp. F-126, for which more substrate-specificity information is available (Watanabe *et al.*, 1989 ▶), is identical with respect to the same three residues forming the substrate carboxyl site. Binding of glycine to this carboxyl-group site will leave its amino group too far away from the cofactor to form a Schiff base. Transamination will require the movement of glycine out of this rigid energetically favourable site, resulting in low activity towards glycine as a donor (0.4% of that of β-alanine; Yonaha *et al.*, 1977 ▶). MBA and l-Ala were modelled into the active site, making an external aldimine link with PLP and orientated for catalysis according to the Dunathan hypothesis (Dunathan, 1966 ▶; Fig. 8 ▶). This positions the cleaved C—H bond of the amino donor normal to the plane of the PLP pyridine ring, pointing towards the active-site Lys288. The side chain of the non-moving PA Phe89 would prevent binding of any amino acid with atoms beyond C^β^, thus explaining the absence of activity towards α-­amino acids larger than alanine. The active site would not bind *R*-α-MBA or d-­Ala in a position favourable for catalysis (Fig. 8 ▶). It appears that l-Ala undergoes the reaction with its carboxyl group not bound to the carboxyl-group site. The methyl (C^β^) group fits the hydrophobic pocket formed by PA Leu60 and PA Phe89, favouring Ala over Gly as an amino donor. Overall, the rigid structure of *Pseudomonas* ωATs severely limits their substrate range.

The related enzyme ω-amino-acid:pyruvate AT from *Pseudomonas* sp. F-126 has been shown to have high activity towards d,l-3-aminobutyrate (Yonaha *et al.*, 1977 ▶). The structure of the substrate-binding site allows us to propose that the observed activity is towards the l-isomer and that *P. aeruginosa* β-A:PyAT can be used for enantioselective catalysis of this substrate.

### 3.11. Substrate-binding site of *C. violaceum* Am:PyAT   

The *C. violaceum* enzyme does not seem to have a fixed substrate carboxyl-binding site. The methyl group of CV Ala425, which occupies the position of PA Gly423, forces the movement of the side chain of CV Trp60 (PA Trp61) by 3 Å into the active site, which effectively blocks the carboxyl site observed in the *Pseudomonas* enzyme. The *C. violaceum* enzyme also has the CV Val423 residue in place of the carboxyl-binding site PA Gln421. The carboxyl group of gabaculine forms a salt bridge with the side chain of CV Arg416 in the *C. violaceum* enzyme structure. The mobility of this CV Arg416 and the flexibility of the loop CV 81–93 allow *C. violaceum* Am:PyAT to accept α-amino acids as protein donors (Kaulmann *et al.*, 2007 ▶).

The aromatic ring of mCPP is positioned in a hydrophobic pocket formed by residues CV Trp60, CV Tyr153, CV Ala231, CV Ile262, CV Leu59 and CV His318 from the adjacent subunit. The active-site lysine is displaced away from the cofactor site to where it was in the apoenzyme structure, with the C^α^—C^β^ bond rotated by 90° away from the position that it occupies in the holoenzyme structure (Fig. 7 ▶ *a*).

The orientation of the mCPP gabaculine ring and its position in the *C. violaceum* enzyme complex differ from those in the *Pseudomonas* ωAT structure (Fig. 7 ▶ *c*). In addition, residues PA Phe89 and PA Phe24 are within close proximity of the mCPP inhibitor in the *P. aeruginosa* AT complex structure. These residues are conserved in the *C. violaceum* AT structure (CV Phe22 and CV Phe88), but in the complex structure they are displaced away from the active site (Figs. 7 ▶ *a* and 7 ▶ *b*).

### 3.12. Extra PLP-binding site   

The structure of gabaculine-inhibited *P. aeruginosa* β-­A:PyAT contained an additional cofactor-binding site which was modelled with full occupancy in all four subunits. The PLP is located on the external surface of the large domain, approximately 17 Å from the nearest active-site PLP atom, with its phosphate group binding to the main-chain N atoms of PA Gly240 and PA Gln243. In the holoenzyme structure of *P. aeruginosa* β-A:PyAT no density was observed at this extra cofactor-binding site, with the side chain of PA Gln243 partially occupying the site. The cofactor excess used in the crystallization of holo *P. aeruginosa* β-A:PyAT was 50 µ*M*, which is about 0.25 PLP molecules per extra site. No density for the cofactor was observed at this site in any of the *C. violaceum* ωAT structures, although both the holoenzyme and the mCPP complex were crystallized in the presence of a large excess of PLP. As no significant conformational changes between holoenzyme and complex structures of *P. aeruginosa* β-A:PyAT occur in this region, we attribute the PLP binding at this additional site to the greater excess of PLP (1 m*M*, five molecules of PLP per protein subunit) used in the crystallization of the *P. aeruginosa* β-A:PyAT–gabaculine complex.

## 4. Conclusions   

The work presented in this paper has provided a structural understanding of the differences in substrate specificity between two industrially important ωATs from *Chromobacterium* and *Pseudomonas* species. Initial determination of the holo structures of both enzymes and the apo structure of *C. violaceum* Am:PyAT did not provide sufficient information to explain why the *Pseudomonas* enzyme shows activity towards the amino donor β-alanine, whilst the *Chromobacterium* enzyme does not. Both enzymes show activity towards the amino donor MBA. Elucidation of the inhibitor-bound mCPP complexes of both enzymes has provided an explanation regarding their substrate specificity and cofactor-binding properties. This information is of significant interest for the application of these enzymes in commercial biocatalysis.

The *C. violaceum* enzyme has low affinity for its cofactor, which is consistent with the structural rearrangements that are observed during catalysis. This also gives the enzyme the unusual property of being only partially inhibited by gabaculine (Schell *et al.*, 2009 ▶). The conformational changes that are observed in the *C. violaceum* enzyme structure upon inhibitor binding are different from those observed on cofactor binding in the same region of the protein. In the inhibitor-bound structure the enzyme is conformationally relaxed in a state between the apoenzyme and holoenzyme structures. The changes observed result in important structural rearrangements in the active-site cavity. Additionally, a significant loop rearrangement results in Leu59 and Trp60 inverting their positions at the bottom of the substrate-binding pocket. Movements in this region have not previously been observed between the *C. violaceum* apoenzyme and holo­enzyme structures.

The flexibility of the *Chromobacterium* enzyme and the absence of a fixed substrate carboxyl-binding site extends its substrate range and increases its applications in the pharmaceutical industry. However, the flexible structure provides no fixed position for the ω-carbon of β-alanine. This feature, coupled with the mobility of the active-site lysine, renders this enzyme inactive towards this amino donor.

The apparent rigidity of the *Pseudomonas* β-A:PyAT scaffold and the defined fixed carboxyl-binding site at a set distance from the cofactor makes this enzyme very active towards β-alanine; however, it significantly restricts its substrate range. In both aminotransferase enzymes the hydrophobic interactions in the substrate pocket orientate MBA in a favourable conformation for trans­amination.

These studies have increased our fundamental understanding of how subtle changes in the structural properties of different AT enzymes have occurred during evolution to catalyse many different reactions in normal cellular metabolism. This knowledge opens the possibility for rational engineering of these enzymes to optimize their use for specific industrial applications.

## Supplementary Material

PDB reference: *P. aeruginosa* β-A:PyAT, holo, 4b9b

PDB reference: gabaculine complex, 4b98

PDB reference: *C. violaceum* Am:PyAT, apo, 4ba4

PDB reference: holo, 4ah3

PDB reference: gabaculine complex, 4ba5

## Figures and Tables

**Table 1:** Summary of data-processing and refinement statistics Values in parentheses are for the outer resolution shell. The Wilson *B* factor was estimated by *SFCHECK* (Vaguine *et al.*, 1999 ▶). Ramachandran plot analysis was performed using *PROCHECK* (Laskowski *et al.*, 1993 ▶).

	*C. violaceum* Am:PyAT			*P. aeruginosa* -A:PyAT	
Crystal	Apoenzyme	Holoenzyme	Complex	Holoenzyme	Complex
Space group	*P*1	*P*1	*P*1	*P*2_1_	*P*2_1_2_1_2_1_
Unit-cell parameters (, )	*a* = 58.8, *b* = 61.9, *c* = 63.9, = 71.9, = 111.3, = 74.6	*a* = 61.9, *b* = 62.2, *c* = 119.6, = 75.1, = 81.7, = 76.2	*a* = 58.5, *b* = 60.6, *c* = 61.3, = 68.4, = 76.2, = 84.3	*a* = 80.4, *b* = 133.2, *c* = 162.0, = 91.8	*a* = 119.2, *b* = 192.5, *c* = 77.3
No. of chains per asymmetric unit	2	4	2	8	4
Wavelength ()	1.54	1.22	1.49	0.92	0.98
Resolution range ()	151.73 (1.821.73)	261.57 (1.661.57)	41.71.76 (1.861.76)	421.64 (1.681.64)	711.65 (1.691.65)
Completeness (%)	90.7 (81.1)	94.4 (90.9)	88.9 (63.7)	99.0 (93.8)	98.9 (99.6)
Multiplicity	5.9 (3.4)	2.0 (2.0)	2.1 (2.1)	4.1 (4.0)	5.0 (5.2)
*I*/(*I*)	25.1 (3.4)	7.1 (1.9)	7.0 (2.0)	19.3 (2.0)	11.4 (2.5)
*R*_merge_ † (%)	5.6 (25.1)	10.2 (29.5)	6.9 (26.0)	10.7 (67.9)	7.2 (65.6)
*R*_cryst_ ‡ (%)	17.0	22.2	17.6	17.5	22.0
*R*_free_ (5% of total data) (%)	21.8	27.3	23.5	21.9	26.0
R.m.s.d. bond lengths§ ()	0.008 [0.019]	0.011 [0.019]	0.010 [0.019]	0.017 [0.019]	0.008 [0.019]
R.m.s.d. bond angles§ ()	1.24 [1.95]	1.46 [1.95]	1.36 [1.95]	1.71 [1.96]	1.28 [1.96]
Wilson *B* factor (^2^)	36.0	28.7	35.2	23.1	26.8
Average *B* factor (^2^)					
Protein	30.8	27.6	31.8	15.0	21.5
Solvent	40.1	33.8	37.2	27.6	29.7
Ligand		21.7	23.2	16.9	25.9
Occupancy of cofactor/inhibitor		LysPLP Schiff base, 1.0	mCPP, 0.6, 0.7	PLP, 0.450.63; LysPLP, 0.160.28	mCPP, 0.91.0
Ramachandran plot analysis, residues in (%)					
Most favoured regions	89.9	88.1	88.4	86.5	88.1
Generously allowed regions	0.6	0.3	1.0	0.3	0.3
Disallowed regions	0	0.4	0.1	0.6	0.5

† *R*_merge_ = 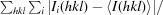 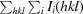, where *I*(*hkl*) is the intensity of reflection *hkl*, 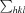 is the sum over all reflections and 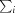 is the sum over *i* measurements of the reflection.

‡ *R*_cryst_ = 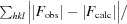 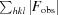.

§ Target values are given in square brackets.

## References

[bb1] Arndt, U. W. & Wonacott, A. J. (1977). Editors. *The Rotation Method in Crystallography* Amsterdam, New York: Elsevier.

[bb2] Braunstein, A. E. & Shemyakin, M. M. (1953). *Biokhimia*, **18**, 393–411. 13219089

[bb3] Burnett, G., Yonaha, K., Toyama, S., Soda, K. & Walsh, C. (1980). *J. Biol. Chem.* **255**, 428–432. 7356625

[bb4] Chen, C. (2008). *Curr. Med. Chem.* **15**, 2173–2191. 10.2174/092986708785747625 18781943

[bb5] Cho, B.-K., Park, H.-Y., Seo, J.-H., Kim, J., Kang, T.-J., Lee, B.-S. & Kim, B.-G. (2008). *Biotechnol. Bioeng.* **99**, 275–284. 10.1002/bit.21591 17680656

[bb6] Cowtan, K. (2010). *Acta Cryst.* D**66**, 470–478. 10.1107/S090744490903947X PMC2852311 20383000

[bb7] DeLano, W. L. (2002). *PyMOL* http://www.pymol.org.

[bb8] Dunathan, H. C. (1966). *Proc. Natl Acad. Sci. USA*, **55**, 712–716. 10.1073/pnas.55.4.712 PMC224217 5219675

[bb9] Emre, M. *et al.* (2004). *New Engl. J. Med.* **351**, 2509–2518. 10.1056/NEJMoa041470 15590953

[bb10] Emsley, P., Lohkamp, B., Scott, W. G. & Cowtan, K. (2010). *Acta Cryst.* D**66**, 486–501. 10.1107/S0907444910007493 PMC2852313 20383002

[bb11] Evans, P. (2006). *Acta Cryst.* D**62**, 72–82. 10.1107/S0907444905036693 16369096

[bb12] Fu, M. & Silverman, R. B. (1999). *Bioorg. Med. Chem.* **7**, 1581–1590. 10.1016/s0968-0896(99)00081-4 10482450

[bb13] Fuchs, M., Koszelewski, D., Tauber, K., Kroutil, W. & Faber, K. (2010). *Chem. Commun.* **46**, 5500. 10.1039/c0cc00585a 20461261

[bb14] Höhne, M., Schätzle, S., Jochens, H., Robins, K. & Bornscheuer, U. T. (2010). *Nature Chem. Biol.* **6**, 807–813. 10.1038/nchembio.447 20871599

[bb15] Humble, M. S., Cassimjee, K. E., Håkansson, M., Kimbung, Y. R., Walse, B., Abedi, V., Federsel, H. J., Berglund, P. & Logan, D. T. (2012). *FEBS J.* **279**, 779–792. 10.1111/j.1742-4658.2012.08468.x 22268978

[bb16] Hwang, B.-Y., Ko, S.-H., Park, H.-Y., Seo, J.-H., Lee, B.-S. & Kim, B.-G. (2008). *J. Microbiol. Biotechnol.* **18**, 48–54. 18239415

[bb17] Ingram, C. U., Bommer, M., Smith, M. E., Dalby, P. A., Ward, J. M., Hailes, H. C. & Lye, G. J. (2007). *Biotechnol. Bioeng.* **96**, 559–569. 10.1002/bit.21125 16902948

[bb18] Isupov, M. N., Antson, A. A., Dodson, E. J., Dodson, G. G., Dementieva, I. S., Zakomirdina, L. N., Wilson, K. S., Dauter, Z., Lebedev, A. A. & Harutyunyan, E. H. (1998). *J. Mol. Biol.* **276**, 603–623. 10.1006/jmbi.1997.1561 9551100

[bb19] Isupov, M. N. & Lebedev, A. A. (2008). *Acta Cryst.* D**64**, 90–98. 10.1107/S0907444907053802 PMC2394828 18094472

[bb20] Iwasaki, A., Yamada, Y., Kizaki, N., Ikenaka, Y. & Hasegawa, J. (2006). *Appl. Microbiol. Biotechnol.* **69**, 499–505. 10.1007/s00253-005-0002-1 16003558

[bb22] Jung, M. J. & Seiler, N. (1978). *J. Biol. Chem.* **253**, 7431–7439. 701263

[bb23] Kabsch, W. (2010). *Acta Cryst.* D**66**, 125–132. 10.1107/S0907444909047337 PMC2815665 20124692

[bb24] Käck, H., Sandmark, J., Gibson, K., Schneider, G. & Lindqvist, Y. (1999). *J. Mol. Biol.* **291**, 857–876. 10.1006/jmbi.1999.2997 10452893

[bb25] Kaulmann, U., Smithies, K., Smith, M. E., Hailes, H. C. & Ward, J. M. (2007). *Enzyme Microb. Tech.* **41**, 628–637.

[bb26] Kim, K.-H. (1964). *J. Biol. Chem.* **239**, 783–786. 14154456

[bb27] Kim, D. S., Moses, U. & Churchich, J. E. (1981). *Eur. J. Biochem.* **118**, 303–308. 10.1111/j.1432-1033.1981.tb06402.x 7285925

[bb28] Laskowski, R. A., MacArthur, M. W., Moss, D. S. & Thornton, J. M. (1993). *J. Appl. Cryst.* **26**, 283–291.

[bb29] Lebedev, A. A. & Isupov, M. N. (2012). *CCP4 Newsl.* **48**, contribution 11.

[bb30] Lebedev, A. A., Young, P., Isupov, M. N., Moroz, O. V., Vagin, A. A. & Murshudov, G. N. (2012). *Acta Cryst.* D**68**, 431–440. 10.1107/S090744491200251X PMC3322602 22505263

[bb31] Leslie, A. G. W. & Powell, H. R. (2007). *Evolving Methods for Macromolecular Crystallography*, edited by R. J. Read & J. L. Sussman, pp. 41–51. Dordrecht: Springer.

[bb32] Malik, M. S., Park, E.-S. & Shin, J.-S. (2012). *Appl. Microbiol. Biotechnol.* **94**, 1163–1171. 10.1007/s00253-012-4103-3 22555915

[bb33] Mann, S., Marquet, A. & Ploux, O. (2005). *Biochem. Soc. Trans.* **33**, 802–805. 10.1042/BST0330802 16042602

[bb34] Mehta, P. K., Hale, T. I. & Christen, P. (1993). *Eur. J. Biochem.* **214**, 549–561. 10.1111/j.1432-1033.1993.tb17953.x 8513804

[bb35] Metzler, D. E., Ikawa, M. & Snell, E. E. (1954). *Biochemistry*, **4**, 1518–1525.

[bb36] Murshudov, G. N., Skubák, P., Lebedev, A. A., Pannu, N. S., Steiner, R. A., Nicholls, R. A., Winn, M. D., Long, F. & Vagin, A. A. (2011). *Acta Cryst.* D**67**, 355–367. 10.1107/S0907444911001314 PMC3069751 21460454

[bb37] Otwinowski, Z. & Minor, W. (1997). *Methods Enzymol.* **276**, 307–326. 10.1016/S0076-6879(97)76066-X 27754618

[bb39] Park, E.-S., Kim, M. & Shin, J.-S. (2012). *Appl. Microbiol. Biotechnol.* **93**, 2425–2435. 10.1007/s00253-011-3584-9 21983703

[bb40] Plosker, G. L. & Figgitt, D. P. (2004). *Pharmacoeconomics*, **22**, 389–411. 10.2165/00019053-200422060-00005 15099124

[bb41] Punta, M. *et al.* (2012). *Nucleic Acids Res.* **40**, D290–D301. 10.1093/nar/gkr1065 PMC3245129 22127870

[bb42] Ramakrishnan, C. & Ramachandran, G. N. (1965). *Biophys. J.* **5**, 909–933. 10.1016/S0006-3495(65)86759-5 PMC1367910 5884016

[bb43] Rando, R. R. (1977). *Biochemistry*, **16**, 4604–4610. 10.1021/bi00640a012 410442

[bb44] Richardson, J. S. (1981). *Adv. Protein Chem.* **34**, 167–339. 10.1016/s0065-3233(08)60520-3 7020376

[bb45] Samsonova, N. N., Smirnov, S. V., Altman, I. B. & Ptitsyn, L. R. (2003). *BMC Microbiol.* **3**, 2.

[bb46] Savile, C. K., Janey, J. M., Mundorff, E. C., Moore, J. C., Tam, S., Jarvis, W. R., Colbeck, J. C., Krebber, A., Fleitz, F. J., Brands, J., Devine, P. N., Huisman, G. W. & Hughes, G. J. (2010). *Science*, **329**, 305–309. 10.1126/science.1188934 20558668

[bb47] Sayer, C., Bommer, M., Isupov, M., Ward, J. & Littlechild, J. (2012). *Acta Cryst.* D**68**, 763–772. 10.1107/S0907444912011274 22751661

[bb48] Sayer, C., Isupov, M. N. & Littlechild, J. A. (2007). *Acta Cryst.* F**63**, 117–119. 10.1107/S1744309107000863 PMC2330129 17277454

[bb49] Schell, U., Wohlgemuth, R. & Ward, J. M. (2009). *J. Mol. Catal. B Enzym.* **59**, 279–285.

[bb50] Schneider, G., Käck, H. & Lindqvist, Y. (2000). *Structure*, **8**, R1–R6. 10.1016/s0969-2126(00)00085-x 10673430

[bb51] Shah, S. A., Shen, B. W. & Brünger, A. T. (1997). *Structure*, **5**, 1067–1075. 10.1016/s0969-2126(97)00258-x 9309222

[bb52] Shin, J.-S. & Kim, B.-G. (1999). *Biotechnol. Bioeng.* **65**, 206–211. 10458742

[bb53] Shin, J.-S., Yun, H., Jang, J.-W., Park, I. & Kim, B.-G. (2003). *Appl. Microbiol. Biotechnol.* **61**, 463–471. 10.1007/s00253-003-1250-6 12687298

[bb54] Toney, M. D., Hohenester, E., Cowan, S. W. & Jansonius, J. N. (1993). *Science*, **261**, 756–759. 10.1126/science.8342040 8342040

[bb55] Vagin, A. & Teplyakov, A. (2010). *Acta Cryst.* D**66**, 22–25. 10.1107/S0907444909042589 20057045

[bb56] Vaguine, A. A., Richelle, J. & Wodak, S. J. (1999). *Acta Cryst.* D**55**, 191–205. 10.1107/S0907444998006684 10089410

[bb57] Watanabe, N., Sakabe, K., Sakabe, N., Higashi, T., Sasaki, K., Aibara, S., Morita, Y., Yonaha, K., Toyama, S. & Fukutani, H. (1989). *J. Biochem.* **105**, 1–3. 10.1093/oxfordjournals.jbchem.a122600 2500426

[bb58] Winn, M. D. *et al.* (2011). *Acta Cryst.* D**67**, 235–242.

[bb59] Winter, G. (2010). *J. Appl. Cryst.* **43**, 186–190.

[bb61] Yonaha, K., Nishie, M. & Aibara, S. (1992). *J. Biol. Chem.* **267**, 12506–12510. 1618757

[bb62] Yonaha, K., Toyama, S. & Kagamiyama, H. (1983). *J. Biol. Chem.* **258**, 2260–2265. 6822556

[bb64] Yonaha, K., Toyama, S., Yasuda, M. & Soda, K. (1977). *Agric. Biol. Chem.* **41**, 1701–1706.

[bb65] Yun, H., Hwang, B.-Y., Lee, J.-H. & Kim, B.-G. (2005). *Appl. Environ. Microbiol.* **71**, 4220–4224. 10.1128/AEM.71.8.4220-4224.2005 PMC1183280 16085806

[bb66] Yun, H., Lim, S., Cho, B.-K. & Kim, B.-G. (2004). *Appl. Environ. Microbiol.* **70**, 2529–2534. 10.1128/AEM.70.4.2529-2534.2004 PMC383019 15066855

